# Syndecan-4 in Tumor Cell Motility

**DOI:** 10.3390/cancers13133322

**Published:** 2021-07-01

**Authors:** Aniko Keller-Pinter, Szuzina Gyulai-Nagy, Daniel Becsky, Laszlo Dux, Laszlo Rovo

**Affiliations:** 1 Department of Biochemistry, Faculty of Medicine, University of Szeged, H-6720 Szeged, Hungary; gyulai-nagy.szuzina@med.u-szeged.hu (S.G.-N.); becsky.daniel@med.u-szeged.hu (D.B.); dux.laszlo@med.u-szeged.hu (L.D.); 2 Department of Oto-Rhino-Laryngology and Head-Neck Surgery, University of Szeged, H-6725 Szeged, Hungary; office.orl@med.u-szeged.hu

**Keywords:** syndecan-4, proteoglycan, migration, EMT, metastasis, cancer, cell polarity, extracellular matrix, actin, calcium, centrosome

## Abstract

**Simple Summary:**

Cell migration is crucial fReaor metastasis formation and a hallmark of malignancy. The primary cause of high mortality among oncology patients is the ability of cancer cells to metastasize. To form metastasis, primary tumor cells must be intrinsically able to move. The transmembrane, heparan sulfate proteoglycan syndecan-4 (SDC4) exhibits multiple functions in signal transduction by regulating Rac1 GTPase activity and consequently actin remodeling, as well as regulating focal adhesion kinase, protein kinase C-alpha and the level of intracellular calcium. By affecting several signaling pathways and biological processes, SDC4 is involved in cell migration under physiological and pathological conditions as well. In this review, we discuss the SDC4-mediated cell migration focusing on the role of SDC4 in tumor cell movement.

**Abstract:**

Syndecan-4 (SDC4) is a ubiquitously expressed, transmembrane proteoglycan bearing heparan sulfate chains. SDC4 is involved in numerous inside-out and outside-in signaling processes, such as binding and sequestration of growth factors and extracellular matrix components, regulation of the activity of the small GTPase Rac1, protein kinase C-alpha, the level of intracellular calcium, or the phosphorylation of focal adhesion kinase. The ability of this proteoglycan to link the extracellular matrix and actin cytoskeleton enables SDC4 to contribute to biological functions like cell adhesion and migration, cell proliferation, cytokinesis, cellular polarity, or mechanotransduction. The multiple roles of SDC4 in tumor pathogenesis and progression has already been demonstrated; therefore, the expression and signaling of SDC4 was investigated in several tumor types. SDC4 influences tumor progression by regulating cell proliferation as well as cell migration by affecting cell-matrix adhesion and several signaling pathways. Here, we summarize the general role of SDC4 in cell migration and tumor cell motility.

## 1. Introduction

Cell migration is a hallmark of tumor cell malignancy and essential for the multistep process of metastasis formation. The capability of invasion and metastasis enables cancer cells to escape the primary tumor mass and colonize new terrain in the body [1]. Beyond its role in metastasis formation and tumor progression, cell motility is essential in a variety of physiological and pathological tasks, such as tissue regeneration, wound healing, angiogenesis, embryonic development, as well as immune cell responses [2]. To form metastasis, primary tumor cells must be intrinsically able to move. These motility mechanisms do not differ from the normal motility cycles [3].

Epithelial-mesenchymal transition (EMT) is defined as the transdifferentiation of epithelial cells into motile mesenchymal cells. EMT occurs during different biological processes, such as embryonic development, tissue regeneration or cancer progression. During EMT, cells acquire enhanced invasion ability, escape from apoptotic signals [4] and gain drug resistance [5]. The epithelial cells maintain cell-to-cell junctions and apico-basal polarity, whereas mesenchymal cells display a motile phenotype and front-rear polarity. The loss of apico-basal polarization and the development of front-rear polarity are characteristic features of EMT. The individual or collective migration of cancer cells require several steps of EMT. For effective single-cell migration, cells must acquire a complete EMT. In contrast, collective cell migration requires a wide spectrum of EMT states: the leader cells gain mesenchymal phenotype, but the follower cells keep the connection with their neighbors with intact cell-cell junctions [6,7].

EMT is controlled by complex signaling pathways, including transcriptional regulation, epigenetic modifications, alternative splicing and modulated by miRNAs, other non-coding RNAs, translational control and post-translational modifications [6,8]. The transforming growth factor beta (TGFβ) signaling is crucial for the induction of EMT, as well as other signaling pathways, including tyrosine kinase receptor signaling [9,10].

**Figure 1:**
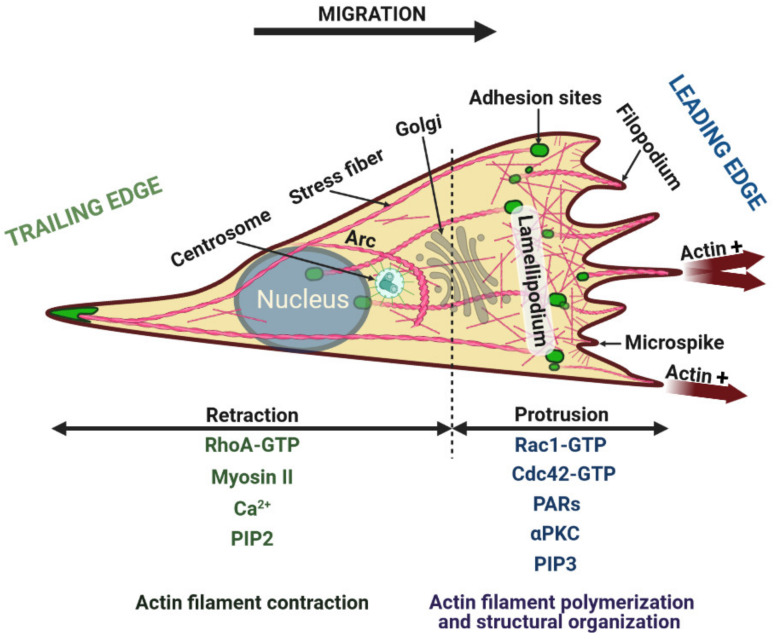
Schematic structure and polarity of a migrating cell in 2D environment during mesenchymal cell migration. Contractile actin bundles (stress fibers) in a migrating cell are represented. Arc-shaped bundles are also observed to move inward under the dorsal cell surface (Arc). At the cell front, in lamellipodia and filopodia, actin filaments are all polarized with their fast-polymerizing ends forwards (for pushing); in the body of the cytoskeleton, actin filaments form bipolar assemblies with myosin to form contractile arrays (for retracting). RhoA: Ras homolog family member A; Ca^2+^: Calcium; Rac1: Ras-related C3 botulinum toxin substrate 1; Cdc42: Cell division control protein 42 homolog; PAR: Partitioning-defective (polarity complex); aPKC: Atypical protein kinase C; PIP2: Phosphatidylinositol 4,5-bisphosphate; PIP3: Phosphatidylinositol 3,4,5-trisphosphate. Image was created with BioRender.com.

The front-rear polarity of migrating cells is developed during the early stages of movement. In 2D environment, migrating cells display flattened morphology, while protrusions of the plasma membrane (i.e., sheet-like lamellipodia and finger-like filopodia) are formed at the cell’s leading edge (Figure 1) [11]. During migration, cell front defines the direction of movement as the tail region forms (Figure 1), causing the morphology of cells to change, forcing them to elongate as a result of actin-cytoskeleton and cell-matrix rearrangement [12]. The shaped tail region is known as the trailing edge, while the front region as the leading edge [13].

Focal adhesions, the cell-matrix contact points, are dynamic, multi-protein structures composed of over 150 proteins [14]. Cell migration requires the continuous assembly and disassembly of focal adhesions, formation of new focal adhesions at the front and disruption at the tail, causing the cell to move [15].

Importantly, migrating cells exhibit different morphologies in vitro in 2D, 3D collagen or 3D cell derived matrix environments [16]. In contrast to the flattened morphology of the cells in 2D, in 3D collagen matrix the migrating cells display a spindle-like phenotype and exhibit multiple small lamellipodia at the leading edge. Moreover, the physical properties of the collagen substrate also affect cancer cell shape both in 2D and 3D [17]. In 3D cell-derived matrix, cells use lobopodial migration and exhibit a more tubular shape with lateral blebs and a leading edge that lacks lamellipodia [18]. The in vitro 1D systems containing matrix fibrils, which usually have a width of 1–2 μm, can closely mimic the biological characteristics of cell migration in 3D matrix, but not on flat 2D substrates [18]. In the 3D living environments, cells exhibit multiple types of migration, such as mesenchymal, amoeboid, lobopodial and collective migration, depending on the local matrix environment [7,19]. All these migration modes are regulated by the local extracellular microenvironment, Rho GTPase signaling and non-muscle myosin contractility [19].

## 2. Cytoskeletal System during Cell Migration

The cytoskeletal system of the mammalian cells is composed of actin network (microfilaments), intermediate filaments and microtubules. The continuous crosstalk between actin, microtubules and intermediate filaments provides their coordinated dynamics to facilitate cell migration [7]. Recently, the septin network was also described as the fourth component of the cytoskeleton. Evidence indicates that all these cytoskeletal systems participate in mammalian cell motility. The roles of the actin network and microtubules in cell motility are well characterized, while less is known about the roles of intermediate filaments and septins.

### 2.1. Rearrangement of the Actin Cytoskeleton during Migration

The dynamic rearrangement of the actin cytoskeleton and cell-matrix interactions is a prerequisite for cell migration [20]. Actin stress fibers play a critical role in in cell adhesion, cell contractility and movement and they are also crucial for preserving and changing the cell’s shape, as well as determining the mechanical properties of the cell surface [21,22]. The main components of these actomyosin contractile stress fibers are the actin microfilaments, myosin II (mechanochemical enzyme) and α-actinin (actin-binding protein) [23]. There are three main types of stress fibers in a migrating cell: ventral stress fibers, transverse arcs and dorsal stress fibers [24]. Ventral stress fibers are associated with focal adhesions at both ends and are located in the tail region of the cells [15]. Transverse arcs are not connected directly to focal adhesions and usually stream back from the anterior edge of the cell toward the center. The dorsal stress fibers are located in the front edge of the cell. They are attached to focal adhesions on the ventral surface of the leading edge and extend dorsally, towards the cell center to bind to transverse arcs (Figure 1). During cell migration, the actin fibers are recycled by a retrograde actin flow process, creating a dynamically active cyclic system [25,26].

Actin polymerization, retrograde actin flow and myosin II-based contractility are all essential for cell migration [27,28]. Cells move by repeating cycles of cell front protrusion and attachment, followed by rear decoupling and retraction. Coordinated polymerization of multiple actin filaments generates protrusive forces that drive plasma membrane protrusion to the cell’s leading edge [29]. Contractile force is generated by myosin motors. Not only the active fibers, but also the cells’ posterior ends are pulled back by this force. Muscle contraction is identical to this process [30,31].

The “dendritic nucleation” is a mechanism of actin turnover in lamellipodia that involves Arp2/3 complex continuously nucleating new actin filaments alongside the pre-existing “primordial” filaments [32,33]. After that, the newly formed filaments elongate and push against the plasma membrane. The diameter of the actin filaments is ~7 nm. They are polar structures, with a plus end (also known as barbed end), where the actin monomers assemble and a minus and (also known as pointed end), where monomers disassemble. The barbed end of the filaments is “capped” after a brief period of elongation; thus, elongation is terminated. Disassembly of the network occurs through a combination of debranching and severing of actin filaments, followed by depolymerization of filament fragments [29]. Overall, the array of branched filaments in lamellipodia undergoes treadmilling by assembling at the front and disassembling throughout its body. Cadherin complexes regulate actin dynamics mainly via α-catenin, which inhibits Arp2/3-mediated branching polymerization [34] and recruits formin, an actin nucleator, to adherent junctions. In addition to their role in providing junctional stability, β-catenin and p120-catenin can act as transcriptional regulators [35]. The key organizers of the actin cytoskeleton dynamics are the members of Rho family of small GTPases [36].

### 2.2. The Role of Intermediate Filaments in Cell Motility

The intermediate filaments are non-polar components of the cytoskeleton with a diameter of 11 nm. The expression of intermediate filaments is tissue specific. During tumor development, changes in intermediate filament expression and composition, such as increases in vimentin levels, are associated with increased invasive capacities [37,38,39]. Vimentin can act as a scaffold for signaling molecules involved in cell motility [40], as well as interact with cell-matrix adhesions [41]. Moreover, vimentin organization modulates the formation of lamellipodia [42]. Keratin intermediate filaments are associated with cell-cell (desmosomal) and cell-matrix (hemidesmosomal) junctions, thereby regulating cell shape, cell adhesion and mechanotransduction [41]. Intermediate filaments exhibit a role in collective migration as well, as keratin filaments control traction forces during collective migration [43,44].

### 2.3. The Complex Function of Microtubules in Cell Migration

Microtubules are dynamic components of the cytoskeleton coordinating cellular migration. They are wider than actin and intermediate filaments with a diameter of 25 nm and composed of α-tubulin and β-tubulin heterodimers. Microtubule assembly is a polarized process and starts from microtubule organizing centers (MTOCs). In most cell the centrosomes serve as a major MTOCs stabilizing the minus ends of microtubules; however, the Golgi complex also participates in microtubule network organization in some cell types [45].

Microtubules are involved in intracellular transport processes, which are crucial for delivery of new membrane components and signaling molecules to the leading edges of migrating cells and the recycling of adhesion receptors (intergrins) [46,47]. The delivery of membranes, mRNAs and polarity factors to the leading edge of a migrating cell supports the formation of protrusions [47,48]. Microtubules also contribute to cell motility through their ability to resist high compressive loads and generate pushing forces to support the formation and maintenance of cell protrusions [46,49]. Moreover, microtubule cytoskeleton controls the formation and maturation of focal adhesions [50] and is also essential for the disassembly of focal adhesions [51].

The microtubule cytoskeleton is an essential regulator of the polarized organization of migrating cells. During cell motility, microtubules display an asymmetric organization, thereby creating a front-rear polarity. By providing pulling forces, they move the nucleus forwards and determine the position of centrosomes [52].

### 2.4. The Role of Septins in Cell Migration

Septins are guanine nucleotide-binding proteins that are highly conserved in eukaryotes and polymerize into hetero-oligomeric complexes, filaments, bundles and rings [53,54]. Septins are recognized as novel components of the cytoskeleton; however, they remain relatively poorly understood compared with other cytoskeletal systems [54]. The septin filaments are formed at the cell cortex or in association with other cytoskeletal components, such as actin or microtubules. By directly associating with cellular membranes, septins are implicated in providing membrane stability, organization of plasma membrane by serving as diffusion barriers for membrane proteins and orientation of cell polarity [54]. Moreover, septins have been shown to function as multimolecular scaffolds by recruiting components of signaling pathway. Growing evidence indicates the role of septins in cell migration. It was reported that septin filaments crosslink actin stress fibers, thereby promoting focal adhesion maturation and cell migration [55].

## 3. Multiple Functions of Rho GTPases in Cell Motility

The Rho family of small GTPases including Rac1 (Ras-related C3 botulinum toxin substrate 1), Cdc42 (Cell division control protein 42 homolog) and RhoA (Ras homolog family member A) are evolutionarily conserved regulators of cell polarity and the actin cytoskeleton [56]. Rho GTPases function as molecular switches alternating between inactive GDP-bound and active GTP-bound forms. The GTP-bound form binds and activates downstream effector proteins, thereby regulating different signaling pathways [57]. The two-state cycle is regulated by three sets of proteins: the guanine nucleotide exchange factors (GEFs), the GTPase-activating proteins (GAPs) and the guanine dissociation inhibitors (GDIs). The GEFs catalyze the exchange of GDP for GTP. GAPs are able to increase intrinsic GTP hydrolysis and are responsible for switching between the active and inactive forms of Rho GTPases. Alternating between GDP- and GTP-bound states may involve cytosol-membrane translocation, as GDIs prevent Rho GTPases from membrane-targeting and activation [58].

Rho GTPases are crucial molecules in the establishment and sustenance of front-rear polarity in migrating cells [59]. Moreover, they play a role in cell division, cell morphology, differentiation and cell migration [60]. Activated Rac1 is enriched along the leading edge (Figure 1), thereby increasing actin polymerization and the formation of lamellipodial membrane protrusions [61]. Rac1 activity decreases towards the tail region of the cell [29,62]. In contrast, RhoA activity is the highest in the tail region (Figure 1) leading to the appearance of contractile actin bundles (stress fibers). RhoA activity also influences the development of mature focal adhesions [63].

The formation of filopodia is regulated by the activation of Cdc42 [63,64]. Both Rac1 and Cdc42 are able to activate the Arp2/3 complex, leading to actin polymerization and the formation of a branched lamellipodial actin network. Cdc42 and Rac1 regulate the polymerization of cortical actin through the members of the Wiskott–Aldrich syndrome protein (WASP)/Scar1 superfamily [65]. The interaction of Cdc42/Rac1 with WASP/Scar proteins unmasks the C-terminal region, thereby mediating the binding of WASP/Scar to the Arp2/3 complex [66]. Arp2/3 complex binds to the sides of preexisting actin filaments and stimulates new filament formation to create branched actin networks [32]. Actin nucleation is induced by Arp2/3 and enhanced by binding of WASP-family carboxyl-terminal domains to the Arp2/3 complex [66]; therefore, the Arp2/3 and WASP proteins act as molecular links for Cdc42 and Rac1 induced cortical actin polymerization [67,68]. Beyond the role of Rho GTPases in the regulation of actin polymerization, they are involved in actin depolymerization as well. Rac1 and RhoA also regulate cofilin activity, thereby affecting actin depolymerization [69].

## 4. Front-Rear Polarity of Migrating Cells

The existence of asymmetry within a cell is referred to cell polarity. The polarization of migrating cells, such as the formation of front-rear edges and the proper orientation of cellular components, is one of the most remarkable conditions for cell movement [15]. Polarity lipids, such as PIP2 (phosphatidylinositol 4,5-bisphosphate) and PIP3 (phosphatidylinositol 3,4,5-trisphosphate) and 3 sets of polarity protein complexes, such as Par (partition defective), Crumbs and Scribble complexes are responsible for the establishment and maintenance of cellular polarity [59,70]. During intracellular polarization of migrating cells, the leading edge is determined by the presence of PIP3, whilst the tail region is determined by PIP2 [59,71] (Figure 1).

The Par polarity complex, composed of Par3, Par6 and atypical protein kinase C (PKC), can determine the front of a migrating cell and the accumulation of Rac1 and Cdc42 [72]. Because these proteins are missing in the back of the cell, the formation of protrusions is inhibited in the rear resulting in directional migration of the cell [15].

During cell migration, actin accumulates in the lamellipodium, thereby creating a front-rear asymmetry within the cell [73]. Polarization of a migrating cell is also defined by the positioning of the cell nucleus and reorientation of the Golgi network and microtubule organizing center towards the leading edge [74,75]. The activity of Rho GTPases is also asymmetrical during migration creating a gradient between the front and the rear of the cell [58,61,63,76,77]. The Rac1 and Cdc42 GTPases exhibit high activity at the front which decreases towards the rear. In contrast, the activity of RhoA is lower at the front and gradually increases towards the trailing edge [63].

In addition, Tiam1, along with the Par polarity complex, facilitates persistent migration through the stabilization of anterior-posterior cell polarization [78]. Par3 interacts with Tiam1, leading to localized Rac1 activation and consequently creating the front-rear gradient of Rac1 and RhoA GTPases in migrating cells [79]. Because Tiam1-mediated Rac1 signaling is required for establishing and maintaining cell polarity [80], the impaired Tiam1 signaling inhibits the formation of front-rear polarization in migrating cells, thereby inhibiting persistent migration.

Migrating cells also create a front-to-back calcium (Ca^2+^) gradient that is essential for cell migration and serves as a coordinator for polarized distribution of molecules [81]. Both Ca^2+^ influx from the extracellular space through different Ca^2+^ channels of the plasma membrane [82] and Ca^2+^ release from intracellular stores (primarily the endoplasmic reticulum) contribute to cytosolic Ca^2+^ concentration [83]. The increasing front–rear Ca^2+^ gradient is involved in the disassembly of focal adhesions and, consequently, the rear end retraction and the movement of the cell. The Ca^2+^ gradient is required to maintain the front–rear polarization of migrating cells by restricting spontaneous lamellipodia formation in the trailing edges [84]. In addition to contractility, changes in intracellular Ca^2+^ affect the activity of calmodulin-dependent enzymes and actin-crosslinking proteins, thus playing a key role in the assembly of adhesions and multilevel junctions [77,85]. High levels of RhoA activity and subsequent actomyosin contractility define the rear of a migrating cell as well as an increased Ca^2+^ concentration and the activation of Ca^2+^-dependent proteases is required to cleave focal adhesion proteins. It was suggested by Tsai et al. that the crosstalk between Ca^2+^ signaling and Rho GTPases would coordinate the oscillations of these factors in the leading edges of migrating cells [86].

## 5. Syndecan Family of Transmembrane Proteoglycans

Syndecans (SDCs) are transmembrane proteoglycans and four family members are distinguished in vertebrates [87]. Due to their transmembrane structure, the most important task of SDCs is to participate in the physical connection and signaling between the extracellular matrix and the cell. SDCs are major mediators of cellular interactions with the pericellular environment, thereby contributing critical functions to cell adhesion receptors. Moreover, they also participate in cell signaling events and numerous biological processes. The expression of SDCs is cell-, tissue- and development-specific. Syndecan-1 (SDC1), also known as CD-138, is expressed in endothelial, epithelial, smooth muscle and plasma cells. Syndecan-2 (SDC2), also known as fibroglycan, is presented mainly in fibroblasts, mesenchymal tissues, whilst syndecan-3 (SDC3) (N-syndecan) is expressed in neurons and developing musculoskeletal system. Syndecan-4 (SDC4, ryudocan), unlike other members of the family, is universally expressed in virtually all cell types in a development state specific manner [87,88,89].

### General Structure of Syndecans

**Figure 2:**
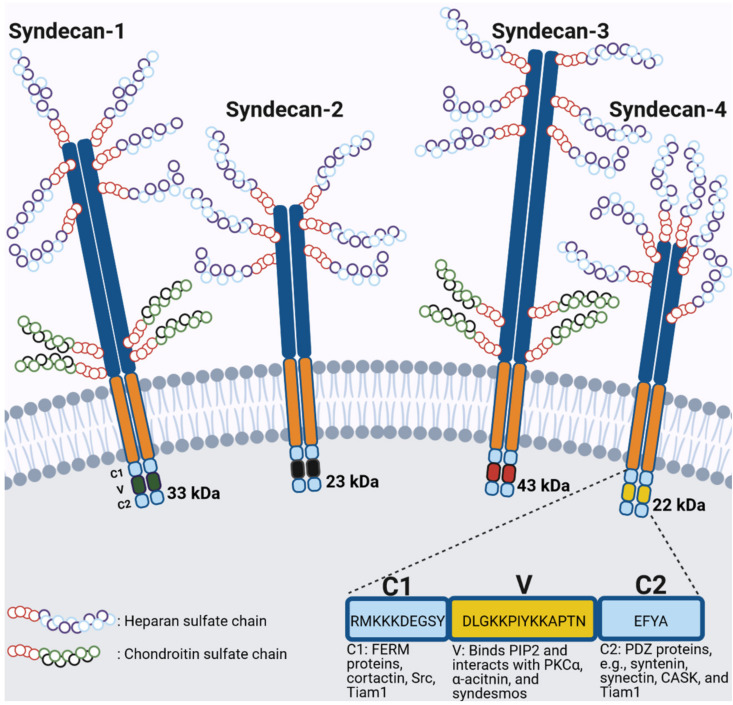
The four-member family of vertebrate syndecans (SDCs). The core proteins of SDC1 and SDC3 are larger than that of SDC2 and SDC4 and can carry both heparan and chondroitin sulfate chains. The glycosaminoglycan chains are attached to the serine residues of the core protein. The cytoplasmic domains are composed of two strongly conserved regions (C1 and C2) separated by an SDC-specific variable (V) region. The main interacting partners of the cytoplasmic domain of SDC4 are shown. PIP2: Phosphatidylinositol-4,5-bisphosphate; PKC: Protein kinase C; PDZ: Postsynaptic density protein; CASK: Calcium/calmodulin-dependent serine protein kinase; Tiam1: T-lymphoma invasion and metastasis-inducing protein 1. Image was created with BioRender.com.

SDCs consist of three domains (Figure 2), an N-terminal, variable extracellular domain (ectodomain), the highly conserved transmembrane domain and the C-terminal intracellular domain [87,90]. Glycosaminoglycan (GAG) side chains are attached to the core protein extracellularly [87,88,91]. Near the N-terminus, heparan sulfate (HS) chains are linked via a tetrasaccharide linker to one of the serine (Ser) residues of the ectodomain by an O-glycosidic bond [92] (Figure 2). Chondroitin sulfate (CS) side chains are also present for SDC1 and SDC3 and bind closer to the transmembrane region [92,93] (Figure 2). The repeating disaccharide of HS is N-acetylglucosamine and uronic acid, which is modified by sulfate and uronic acid epimerization to iduronic acid. The HS chains contain 2-O-sulfated iduronic acid and N-, 6-O, or (rarely) 3-O-sulfated glucosamine subunits. In CS chains, N-, 6-O or 4-O-sulfated acetylgalactosamine subunits are present [94,95].

The extracellular domain has plenty of interacting partners, such as matrix proteins, e.g., fibronectin, matrix metalloproteinases (MMPs), growth factors and cytokines. SDCs can recruit soluble ligands, thereby increasing their local concentration and they can also modulate the ligand-dependent activation of primary signaling receptors at the cell surface [89,96,97], or can protect growth factor precursors from activation [98]. The role of SDCs in tumor cell proliferation was reported in numerous cases. SDC1 drives proliferation through the Wnt/β-catenin pathway in multiple myeloma, but defeats cell growth in colorectal carcinoma via the inhibition of JAK1/STAT and Ras/Raf/MEK/ERK pathways [99]. Moreover, SDC1 is the key mediator of the reactive stromal response that promotes the proliferation of breast cancer cells [100]. SDC2 promotes tumorigenic activity in colon carcinoma cells [101]. SDC4 regulates autotaxin-β induced proliferation in osteosarcoma [102]. The SDC4-α5β1 integrin mediated cell adhesion to fibronectin reduces tumor cell proliferation, whilst the tenascin-C-mediated inhibition of SDC4-fibronectin interaction and consequently the impaired fibronectin-induced signaling enhances the proliferation of glioblastoma cells [103]. The ectodomain of SDCs can also promote the adhesion and penetration of bacteria and viruses [104,105,106,107], the uptake of positively charged cell-penetrating peptides [108], the cell surface binding of cationic poly- and lipoplexes [109], as well as the cellular internalization of lipoplexes [110]. The ectodomain of SDCs can be cleaved by proteolytic enzymes (secretases), such as members of ADAM (disintegrin and metalloproteinase) family and MMPs. This ectodomain shedding also plays a role in pathophysiological processes, including inflammation and tissue regeneration [94,105,111,112].

The transmembrane domain is the most conserved part of the molecule and also shows high similarity within the family. It contains a GXXXG motif that strongly influences the formation of SDS (sodium dodecyl sulfate) resistant dimers [113,114].

The cytoplasmic domain is short and comprises a variable (V) region that is unique for each member of the SDC family and two conserved regions preceding (C1) and following (C2) the V region [91,115]. The C1 region can bind to the members of the FERM (four-point-one, ezrin, radixin, moezin) family, which are membrane- and actin-associated proteins and also binds Src kinase and cortactin [116]. The EFYA motif of the C2 region binds PDZ (postsynaptic density protein) domain containing proteins, such as syntenin, synectin, synbindin, CASK (calcium/calmodulin-dependent protein kinase) or Tiam1 (T-lymphoma invasion and metastasis-inducing protein 1) [91,117,118].

## 6. Structure, Interacting Partners and Signaling of Syndecan-4

SDC4, similarly to other members of the family, is involved in signal transduction processes across the cell membrane. Unlike other SDCs, it is universally expressed and present in virtually all cell types. SDC4 plays a major role in cell proliferation, migration, cell adhesion and it is also involved in cytokinesis, endocytosis and mechanotransduction [88,97,102,119,120,121,122,123]. The extracellular domain binds several growth factors, such as FGF2 (fibroblast growth factor-2) [124], HGF (hepatocyte growth factor) [125], VEGF (vascular endothelial growth factor), PDGF (platelet derived growth factor) [117,126], or the myostatin precursor promyostatin [98] and also different cytokines, like MCP-1 (Monocyte chemoattractant protein-1) [127], or SDF-1 (Stromal cell-derived factor-1, also known as CXCL12) [128]. In addition, extracellular matrix components (e.g., fibronectin), proteases, protease inhibitors are interacting partners, as well. By directly binding to fibronectin, SDC4 is involved in cell adhesion [129], thereby also influencing cell migration.

SDC4 participates in several signaling pathways and functions as a structural protein (Figure 2). The V region of the cytoplasmic domain of SDC4 also binds to PIP2 and activates PKCα [130,131,132,133]. SDC4 dimer forms a tetramer with 2 PIP2 molecules, which binds to the catalytic subunit of PKCα. The resulting activation complex is regulated by the phosphorylation of the cytoplasmic Ser179 (human Ser179, rat Ser183) of SDC4 [134], which alters the conformation of the C2 region of the cytoplasmic domain, leading to loss of PIP2 binding and consequently the lack of PKCα activation [93,135]. PKCα is a Ca^2+^-dependent conventional PKC isoform, but its activation through SDC4 is independent of changes in intracellular Ca^2+^ levels and consequently it is active in the presence of EDTA (ethylenediaminetetraacetic acid) [81].

Moreover, the roles of SDC4 in the regulation of intracellular Ca^2+^ levels were also reported. SDC4 regulates transient receptor potential canonical (TRPCs) channels to control cytosolic Ca^2+^ equilibria, thus consequently cell behavior. SDC4 can recruit PKCα to target serine714 of TRPC7 increasing intracellular Ca^2+^ concentration with a subsequent control of the cytoskeleton in fibroblasts [136]. However, a direct interaction between SDC4 and TRPC7 has not been reported. In contrast, in podocytes, SDC4 knockdown reduced the cell surface expression of TRPC6 channel and reduced the Ca^2+^ concentration [137]. Furthermore, knocking down of SDC4 expression in HaCaT keratinocytes did not affect intracellular Ca^2+^ level, whereas silencing the expression of both SDC1 and SDC4 decreased it by modulating TRPC4 channels [136]. Moreover, the development of intracellular front-to-rear Ca^2+^ gradient is also determined by SDC4 in migrating cells [138]. Knocking down of SDC4 expression decreased cell motility and abrogated Ca^2+^ gradient and centrosome reorientation during migration [138].

SDC4 also establishes contact with the actin cytoskeleton through the biding of SDC4 cytoplasmic domain to α-actinin, a cross-linking protein between actin filaments [131]. SDC4 expression affects the nanoscale structure of the lamellipodial actin network during cell migration. SDC4 knockdown decreased the number of branches as well as the length of branches of the lamellipodial actin cytoskeleton in migrating cells [138].

### 6.1. Syndecan-4 and the Regulation of Rac1/RhoA Activity

SDC4 affects Rac1 activation and accumulates active Rac1 at the leading edges of migrating cells, thus ensuring the formation of membrane extensions [139,140]. The polarized distribution of active Rac1 is essential for directional cell movement. SDC4 knockout fibroblasts migrate randomly as a result of high delocalized Rac1 activity [140].

Tiam1 is a GEF acting as a specific activator for Rac1 [141]. Tiam1 is involved in essential biological processes such as cell migration [57] and cell polarization [141]. Via its relationship with the Arp2/3 complex, Tiam1 regulates actin polymerization and actin cytoskeleton rearrangement [142]. The direct interaction between SDC4 and Tiam1 has been previously demonstrated. SDC4 binds Tiam1 via C2 region of the cytoplasmic domain and the cytoplasmic Ser of SDC4 is also involved in Tiam1 binding [118]. Consequently, SDC4 regulates Tiam1 binding and Rac1 activity in a Ser179 phosphorylation-dependent manner [118]. Moreover, SDC4 also affects the expression and distribution of Tiam1 and influences the persistence of the cell movement in myoblasts [119].

SDC4-dependent binding and activation of PKCα guide PKCα activity to SDC4-regulated membrane microdomains, where PKCα can phosphorylate specific substrates locally. Regulators of the small GTPase RhoA, which facilitates focal adhesion and stress fiber assembly, are potential candidates. RhoA-GTP is necessary for signaling after SDC4 engagement at the cell surface [143], where there is an increase in GTP load and, thus, activity [144]. RhoGDIα (also known as RhoGDI1), which is considered to be phosphorylated, seems to be one of the substrates [144]. The SDC4-dependent activation of RhoA is mediated by PKCα during focal adhesion formation [144]. Moreover, SDC4-mediated Rac1 activation is also controlled by the RhoG activation pathway [145]. SDC4 clustering activates PKCα, which phosphorylates RhoGDI1 at Ser96, thereby triggering the release of RhoG and leading to polarized activation of Rac1 [145].

### 6.2. Syndecan-4 and Focal Adhesion Formation

The formation of α5β1 integrin-dependent focal adhesions requires SDC4 enrichment in focal adhesions [115,143,146,147]. The heparin binding domain of fibronectin binds to the HS side chains of SDC4 [146,148], thereby fibronectin forms a bridge between SDC4 and α5β1 integrins. The binding of fibronectin to HS chains of SDC4 is essential for focal adhesion formation [146,147,148].

During the accumulation of integrins in focal adhesions, focal adhesion kinase (FAK) is autophosphorylated at Tyr397 to serve as a binding site for Src kinase and subsequently phosphorylated on additional tyrosine side chains [149]. Because syndecan-4 regulates the phosphorylation of FAK, the phosphorylation levels of FAK Tyr397 were lower in SDC4 knockout fibroblasts [150].

PKCα activity is required for the formation of mature focal adhesions. PKCα is directly linked to β1 integrins [151]. In this way, the cytoplasmic domain of SDC4 binds to β1 integrin indirectly via PKCα [147,151]. The cytoplasmic domain of SDC4 can also bind to integrin receptors through focal adhesion proteins. The cytoplasmic domain of SDC4 interacts with paxillin through syndesmos [152], which coordinates the organization of focal adhesions. Paxillin can bind to α4 or α9 and β1 integrins directly or indirectly via other focal adhesion proteins such as vinculin and talin [153].

SDC4 interacts directly with α-actinin [130,131]. Because α-actinin binds focal adhesion proteins, such as vinculin and zyxin, the α-actinin binding serves as a link between SDC4, focal adhesions and the cytoskeleton [154]. Moreover, knocking down of SDC4 expression was reported to induce the decoupling of vinculin from F-actin filaments [155]. SDC4 has been identified as a binding partner of dynamin II GTPase via its PH domain and the interaction between dynamin II and SDC4 is important in mediating focal adhesion and stress-fiber formation [156]. Therefore, SDC4 serves as a central mediator in focal adhesion formation by bridging the interactions between integrins, fibronectin and intracellular molecules.

## 7. SDC4 and Tumor Cell Migration

SDC4 contributes to the development and progression of tumors by affecting cell proliferation, invasive growth, migration, metastases formation, or angiogenesis [157,158,159]. SDC4 functions at the cell surface as a signaling interface to affect these processes serving as a co-receptor for soluble ligands, such as growth factors and chemokines and interacting with integrins and growth factor receptors [160].

SDC4 expression is dysregulated in several tumor types, in most cases the tumor cells exhibit SDC4 overexpression [160]. However, it has been also demonstrated, that SDC4 has the potential to act as an anti-migratory/anti-invasive tumor suppressor [161]. SDC4 expression is downregulated in colon carcinoma cells [162] and it is upregulated in normal breast tissue compared to malignant breast tissue [163]. However, SDC4 is overexpressed in melanoma, liver cancer [160], ovarian carcinoma [164], mesothelioma and fibrosarcoma [165]. SDC4 has previously been linked to a high histological grade and a negative estrogen receptor status [166], implying that it may be a predictor of poor prognosis in breast cancer. SDC4-silenced breast carcinoma cells show decreased ability to form bone metastasis in mice [102] and reduced SDC4 expression is associated with reduced metastatic potential in testicular germ cell tumors [167]. Increased SDC4 expression is related to the existence of distant metastasis and increased size of the tumor mass in osteosarcoma [168], but increased patient survival in renal cell carcinoma [169].

Several studies discuss the role of SDCs in EMT. SDC1 is known to inhibit EMT in human oral cancer cells [170]. In contrast, SDC1 mediates EMT in prostate cancer [171] and the expression of SDC1 (and also SDC2) is correlated with EMT markers (E-cadherin, β-catenin) in prostate cancer [172]. SDC2 has a tumorigenic role by promoting EMT in colorectal cancer [173]. Less is known about the role of SDC4 in EMT. SDC4 is known to positively regulate TGFβ1-induced EMT (via Snail) in lung adenocarcinoma cells [174], whilst SDC4-signalling negatively regulates the production of TGFβ1 (reported in the kidneys of SDC4 KO mice) [175]. Moreover, SDC4 silencing is shown to repress EMT in papillary thyroid cancer cells [176].

SDC4 contributes to the regulation of cell motility in various cancer cell types, such as melanoma, breast cancer, lung, or cervical cancer cells (Table 1).

### 7.1. Melanoma

SDC4 has a tumor suppressor property in melanoma. SDC4 silencing increases the migration, whilst SDC4 overexpression decreases the migration of melanoma cells [177,178,179,180]. The tumor suppressor role of SDC4 was also shown in vivo as the overexpression of SDC4 resulted in decreased pulmonary metastatic potential and decreased lymph node metastasis of B16F10 melanoma cells in mice [179]. Similarly, it has been recently shown, that lumican, a small leucine-rich proteoglycan, inhibits in vivo metastasis formation of melanoma [181]. Moreover, syntenin-1 negatively regulates cell migration and SDC4-mediated cytoskeletal organization [179]. FGF2 is essential for the migration of M5 melanoma cells by downregulating FAK Tyr397 phosphorylation during fibronectin-mediated cell adhesion and, thereby promoting cell migration [177]. FGF2 also decreased SDC4 expression in M5 melanoma cells [177]. The matricellular protein cysteine-rich angiogenic inducer 61 (Cyr61) interacts with SDC4, activates integrins and induces metastasis formation, migration and tumorigenicity in MV3 human melanoma cells [178]. Lysophosphatidylcholine (LysoPC) C18:0 decreased the metastatic spread of murine melanoma cells, the cell membrane rigidification by LysoPC C18:0 appears to prevent the formation of focal adhesion [180], which is required for migration and tumor metastasis. Saturated LysoPC activates PKCδ to phosphorylate SDC4 thereby deactivating PKCα and reducing FAK activity [180].

### 7.2. Breast Cancer

The role of SDC4 in breast cancers has not clearly been understood, as we have controversial data regarding the correlation of SDC4 expression and breast cancer prognosis [160,182]. MMPs cleave the extracellular domains of SDCs, which may have a significant role in tumor progression. ADAMTS (a disintegrin and metalloproteinase with thrombospondin motifs)s, a family of secreted proteinases, are involved in the cleavage of proteoglycans. Overexpression of ADAMTSs in cancer cells might be a possible invasive mechanism in order to degrade proteoglycans [183]. ADAMTS-15 decreases the migration of MDA-MB-231 and MCF-7 breast cancer cells in association with the increased cell surface expression of SDC4 [184]. This effect of ADAMTS-15 is not linked to its metalloproteinase function [184]. Moreover, silencing of SDC4 expression rescued the effect of ADAMTS-15 on cell motility in breast cancer cells [184]. SDC4 silencing decreases EGF-mediated chemokinesis and human epidermal growth factor receptor 1 (HER1, also known as EGFR)-induced migration of MCF10A human mammary gland epithelial cells [185]. Overexpression of SDC4 decreases the invasion of breast adenocarcinoma cells into 3D collagen matrix, whilst SDC4 silencing increases the invasiveness. SDC4 inhibits cell invasion, whilst K-Ras-induced α2β1 integrin and membrane type-1 matrix metalloproteinase (MT1-MMP) promote this function. The mutational activation of K-Ras increases the expression of all these proteins suggesting a complex regulatory mechanism of tumor cell invasiveness and metastasis formation [186].

The antimicrobial peptide LL-37 promotes the migration of breast cancer cells via PI3K/AKT signaling and increases intracellular Ca^2+^ levels via Transient Receptor Potential Cation Channel Subfamily V Member 2 (TRPV2). Because the silencing of SDC4 expression decreased LL-37-induced migration and decreased Ca^2+^ influx, SDC4 is essential for both functions of LL-37 [187]. Moreover, by its GAG chains, SDC4 is crucial for LL-37 binding to the cell surface [187]. The Ca^2+^-binding protein S100A4 and its interacting partner, Ca^2+^-dependent protein crosslinking enzyme tissue transglutaminase (TG2), promote tumor cell migration. S100A4 directly interacts with SDC4 and increases the expression of SDC4, whilst recombinant SDC4 administration inhibits the migration of R37 rat mammary cells by competing with the cell surface SDC4 [188]. The SDC4-α5β1 integrin signaling through PKCα participates in TG2/S100A4-mediated tumor cell migration [188]. The branched peptide NT4 exhibits antagonist binding to the GAG chains of HS proteoglycans. NT4 binds SDC4, thereby target cancer cells and inhibit their migration and FGF-induced invasion [189].

Estrogen receptor signaling plays a critical role in the development and progression of hormone-dependent breast cancer. Estradiol (E2) decreases the expression of SDC4 and insulin-like growth factor receptor (IGFR) regulates the expression of SDC4 in the presence, as well as in the absence of E2 [190]. The proteoglycan lumican is known to play a role in estrogen-mediated functions of breast cancer cells, including EMT. Lumican downregulates integrin signaling (FAK, Erk1/2, AKT) [191] and inhibits EMT and the formation of lamellipodia in breast cancer cells [192].

### 7.3. Lung Cancer

SDC4 participates in tumor growth as the size of lung carcinoma tumors was reduced in SDC4 KO mice [193]. Increased levels of SDC4 expression were found in response to lung injury [194], as well as after tumor cell seeding [195]. Moreover, the cell surface expression of SDC4 is regulated by ADAMTS-1 via MMP9 and SDC4 (together with ADAMTS-1) inhibits migration of lung endothelial cells [126]. Cell migration is also inhibited by the interaction of SDC4 and the antifibrotic chemokine CXCL10 in primary lung fibroblasts [194]. In contrast, SDC4 promotes cell migration and invasion of A549 lung adenocarcinoma cells both in wound healing and chemotaxis assays and SDC4 positively regulates TGFβ1-mediated EMT via Snail [174]. The proteolytic shedding of SDCs leads to the release of the soluble N-terminal ectodomain from a transmembrane C-terminal fragment (tCTF). The transmembrane C-terminal fragment (tCTF) of SDC4 increased in vitro migration (examined in wound scratch assay) of SDC1-deficient A459 cells equivalently to that of SDC1 tCTF, whilst the presence of the tCTF of SDC1 was sufficient for the lung metastasis formation in vivo [196].

### 7.4. Other Tumor Types

SDC4 contributes to the regulation of cell migration in numerous cancer cell types and several extracellular modulators of this process are identified. The chemokine SDF-1, also known as C-X-C motif chemokine 12 (CXCL12), binds to SDC4 thereby regulating migration and invasion of choriocarcinoma cells [197]. Moreover, SDC4 is essential for CXCL12-induced migration and invasion of hepatoma cells [198] and human cervix carcinoma (HeLa) cells [199]. The extracellular calumenin decreases HeLa cell migration via SDC4 and α5β1-integrin-dependent suppression of ERK1/2 signaling [200]. Human epidermal receptor 1 (HER1), also known as epidermal growth factor receptor (EGFR), induces cell invasion of skin squamous cancer cells via SDC4-dependent activation of α6β4 integrins [185]. Silencing of SDC4 expression decreases migration and invasion of papillary thyroid cancer cells and inhibits epithelial-mesenchymal transition via Wnt/beta-catenin pathway [176]. SDC4 is also involved in the RANTES/CCL5 signaling and is necessary in RANTES/CCL5-induced invasion and migration of hepatoma cell lines [201].

**Table 1:** Overview of SDC4-dependent migration and SDC4 expression in different tumor cell models.

Cell Type	Migration Assay	Signaling Pathway	Biological Effect	Citation
4T1 and MDA-MB-231 breast cancer cells	-	-	SDC4 has an anti-migratory, anti-invasive tumor suppressor role.	[161]
Colon carcinoma cells	-	SDC4 expression	SDC4 is downregulated in colon carcinoma cells.	[162]
Infiltrating breast carcinoma tissues	-	SDC4 expression	SDC4 is upregulated in normal breast tissue compared to malignant breast tissue	[202]
Human ovarian carcinoma cell line NIH:OVCAR5	Modified Boyden chamber chemotaxis, Matrigel invasion assay	Carbohydrate modifications	The migration, invasion and tumor growth of ovarian carcinoma is mediated by the carbohydrate modifications of proteoglycans.SDC4 is upregulated in ovarian carcinoma.	[164]
Mesothelioma, fibrosarcoma	-	SDC4 expression	SDC4 is upregulated in mesothelioma and fibrosarcoma.	[165]
Breast carcinoma samples from patients	-	SDC4 expression	SDC4 is associated with high histological grade and a negative estrogen receptor status in breast carcinoma.	[166]
4T1 mouse breast cancer cells	-	bone metastasis formation	SDC4-silenced breast carcinoma cells have decreased ability to form bone metastasis in mice.	[102]
JKT-1 human seminoma cell line, NTERA-2 human embryonal carcinoma cell line, NCCIT teratocarcinoma cell line	-	SDC4 expression—metastatic potential	Reduced SDC4 expression is associated with reduced metastatic potential in testicular germ cell tumors.	[167]
Patients with primary high grade intramedullary osteosarcoma, with low grade central osteosarcoma, with osteoid osteoma and normal bone tissues	-	SDC4 expression—metastasis formation, tumor size	Increased SDC4 expression is associated with the formation of distant metastasis and increased tumor size in osteosarcoma.	[168]
Renca (mouse), 786-O and Caki-2 (human) renal carcinoma cells	Wound scratch assay, Transwell assay	High SDC4 expression in renal cell carcinoma	High SDC4 expression determines increased patient survival in renal cell carcinoma.	[169]
M5 human metastatic melanoma cells	Chemotaxis assay, wound scratch assay	FGF-2/SDC4	FGF-2 regulates melanoma cell migration in a SDC4-dependent manner.	[177]
MV3 human melanoma cell line	Wound scratch assay	Cyr61/SDC4	Cyr61 is exocytosed by binding to SDC4. Cyr61 binds to and activates integrins, thus induce migration, metastasis formation and tumorigenicity.	[178]
Rat embryonic fibroblasts (REFs), A375 melanoma cells, B16F10 melanoma cells, C57BL/6 mice	Transwell migration assay, lung metastasis model	Syntenin-1/SDC4 SDC4—inhibition of cancer-associated melanoma migration	SDC4 overexpression decreases melanoma cell migration in vitro and reduces the metastatic potential of melanoma in vivo. Syntenin-1 negatively regulates SDC4-mediated inhibition of cell migration and SDC4-mediated tumor suppression in melanoma.	[179]
B16.F10 murine melanoma cells	Wound scratch assay	LysoPC/PKCδ/SDC4/PKCα/FAK	LysoPC C18:0 decreases the metastatic spread of melanoma cells. LysoPC activates PKCδ to phosphorylate SDC4 thereby deactivating PKCα and reducing FAK activity.	[180]
MDA-MB-231 and MCF7 human breast cancer cells	2D: wound scratch assay 3D: Matrigel and Collagen Type I	ADAMTS-15/SDC4	Inhibition of mammary cancer cell migration by ADAMTS-15 requires SDC4.	[184]
Human HaCat keratinocytes, A431 (human squamous skin epithelial) carcinoma cells, MCF10A (human mammary gland epithelial) cells	Wound scratch assay	HER1(EGFR)/α6β4 integrin/SDC4	HER1-dependent activation of α6β4 integrin and α6β4 integrin-mediated cell invasion require SDC4.	[185]
MDA-MB-231 breast adenocarcinoma cells	Cell invasion into 3D collagen gel	Integrin α2β1/MT1-MMP/SDCs–K-Ras mutant cell invasion	K-Ras mutant cells show increased expression of SDC1 and SDC4. MT1-MMP and α2β1 integrin promote invasive phenotype, SDCs reduce invasion into collagen matrices.	[186]
MCF7, MDA-MB-435s and MDA-MB-231 breast cancer cells	Migration chamber (insert with polyethylene filter with 8 µM pores)	LL-37/SDC4LL-37/TRPV2/ic. Ca^2+^ LL-37/PI3K/AKT/motility	SDC4 is a receptor for LL-37 increasing Ca2+ levels via TRPV2 channels and increasing the motility of breast cancer cells via PI3K/AKT signaling.	[187]
Non-metastatic rat mammary R37 cells, highly metastatic KP1 cells (R37 cells transfected with S100A4)	Wound scratch assay	SDC4/α5β1 integrin/PKCα—TG2 and S100A4-mediated cell migration	S100A4 mediates migration of tumor cells via SDC4 and α5β1 integrin-mediated PKCα activation.	[188]
PANC-1 human pancreas adenocarcinoma cells, HT-29 human colon adenocarcinoma cells, MCF-7 and MDA-MB-231 human breast adenocarcinoma cells	-	NT4—SDC4	The branched peptide NT4 inhibits cancer cell migration and FGF-induced invasion. NT4 binds to SDC4, the expression of SDC4 is upregulated breast cancer cells.	[189]
MCF-7 (low metastatic ERa+), MDA-MB-231 (highly invasive ERa-) breast cancer cells	Wound scratch assay	IGFR/SDC4 expression	IGFR regulates the expression of SDC4 both in the presence and in the absence of E2 in breast cancer cells.IGFR inhibitors reduced the migration of MCF-7 cells but did not have a significant effect on MDA-MB-231 cells.	[190]
C57Bl/6 mouse primary lymphatic endothelial cells, Lewis lung carcinoma cells, bone marrow–derived DCs (BMDCs)	Transwell migration assay, in vivo migration assay (BMDCs migration into lymph node), tumor growth studies	SDC4—dendritic cell maturation	SDC4-deficient mice exhibit impaired tumor growth and increased infiltration by mature dendritic cells. SDC4 is the dominant proteoglycan on dendritic cells.	[193]
Primary lung fibroblasts	Boyden chamber, chemotaxis assay	CXCL10—SDC4	In response to lung injury, the expression of SDC4 is increased. SDC4 directly interacts with CXCL10 and they inhibit the migration of fibroblasts. SDC4 is required for the inhibitory effect of CXCL10 during fibrosis.	[194]
Human blood–derived monocytes, primary pulmonary endothelial cells, Lewis lung carcinoma cells (LLC1)	Boyden chamber, Transwell assay, spontaneous metastasis in mice	-	Increased expression of SDC4 is observed in endothelial cells after tumor cell seeding to the lungs.	[195]
Mouse lung endothelial cells	Random migration assay; ex vivo C57BL/6 mice aortic ring assay	ADAMTS-1—MMP9—SDC4	ADAMTS-1 modulates the cell surface expression of SDC4 via MMP9. ADAMTS-1 and SDC4 inhibit cell migration, whilst their inhibition increase angiogenesis.	[126]
A549 human lung adenocarcinoma cells	Wound scratch assay, transwell chemotaxis assay	SDC4/Snail/TGFβ1-induced EMT	SDC4 promotes migration and invasion of lung adenocarcinoma cells. SDC4 positively regulates TGFβ1-induced EMT (via Snail), consequently promoting a more motile phenotype.	[174]
A549 lung tumor epithelial cells	Wound scratch assay, matrigel invasion assay, in vivo lung tumor metastasis	ADAM17–SDC4 cleavage;SDC1—in vivo lung tumor metastasis	SDC1 tCFT was sufficient to induce lung metastasis formation in SCID mice, whilst SDC4 tCFT achieved as efficient wound closure as SDC1 tCFT. (tCTF = transmembrane C-terminal fragment)	[196]
JAR choriocarcinoma cells	Modified Boyden-chamber chemotactic assay	CXCL12/SDC4	SDC4 binds to CXCL12 and regulates CXCL12-mediated cell migration and invasion. SDC4 plays a role in the invasiveness of extravillous cytotrophoblast in moles.	[197]
Huh7 human hepatoma cells	Bio-coat cell migration chambers, Matrigel invasion assay	SDF-1 (CXCL12)/ CXCR4/SDC4	SDC4 is essential for SDF-1 (CXCL12) induced migration and invasion of hepatoma cells.	[198]
Human cervix epitheloid carcinoma (HeLa) cells	Bio-coat cell migration chambers, Matrigel invasion assay	SDC4–SDF-1/CXCL12– PKCδ, JNK/SAPK	SDC4 plays a role in SDF-1/CXCL12-mediated cell invasion and chemotaxis. PKCδ and c-jun NH2-terminal kinase/stress-activated protein kinase (JNK/SAPK) are involved in the SDF-1/CXCL12-induced cell invasion.	[199]
Human cervix epitheloid carcinoma (HeLa) cells	Wound scratch assay, Transwell assays	Calumenin–FN, SDC4, α5β1 integrin–ERK1/2	Calumenin inhibits cell migration and tumor metastasis through FN, SDC4 and α5β1-integrin by the suppression of ERK1/2 signaling.	[200]
Papillary thyroid cancer cells K1, BCPAP, TPC-1 and IHH-4, normal thyroid Nthy-ori3-1 cells	Transwell assay, wound scratch assay	SDC4—Wnt/β-catenin signaling pathway	SDC4-silencing decreased papillary thyroid cancer cell migration and invasion and represses EMT. Furthermore, SDC4-silencing suppresses Wnt/βcatenin signaling, thus promoting apoptosis.	[176]
Huh7, HepG2and Hep3B human hepatoma cells	Bio-coat migration chambers, Matrigel invasion assay	RANTES/CCL5—SDC4	SDC4 is essential in RANTES/CCL5-mediated hepatoma cell invasion and migration and its binding to the cell plasma membrane.	[201]

## 8. Syndecan-4 and Non-Cancer Cell Migration

Beyond the role of SDC4 in tumor cell migration, SDC4 was shown previously to affect migration in various non-cancerous cell types as well, including fibroblasts [140], myoblasts [119,138], endothelial cells [203], or hepatic stellate cells [204]. SDC4 may also contribute to arthritis development by affecting the migration of B-cells [205] and the pathogenesis of preeclampsia by modulating trophoblast migration [206]. SDC4 is necessary for the maturation of dendritic cells, which requires a switch in SDC expression and the elevated level of SDC4 ensures the increased motility of the cells and their relocation to the lymphoid tissues [207]. The monitoring of intestinal wound healing in SDC4 KO mice revealed that SDC4 is necessary for wound closure both in vitro and in vivo [208]. Moreover, in vivo wound healing assays of myofibroblasts indicate that SDC4 is important for the proper cardiac functions after myocardial infarction as it is a crucial mediator of granulation tissue formation thereby preventing cardiac rupture [209]. Because SDC4 KO mice also exhibit impaired angiogenesis, SDC4 may affect angiogenesis by the modulation of endothelial cell migration [210]. Administration of SDC4 proteoliposomes intensified the proliferation, migration and angiogenic tube formation of endothelial cells [211].

Shin et al. reported that SDC4 overexpression increased the migration of turkey satellite cells and increased the activation of RhoA GTPase and these phenomena required the cytoplasmic domain of SDC4 [212]. Other studies observed reduced motility after SDC4 knockdown in different cell types (hepatic stellate cells [204], lens epithelial cells [213], human umbilical vein endothelial cells (HUVECs) [210] and dendritic cells [207]), consistent with our observations [119,138], whereas high SDC4 level promoted migration [174,204,206].

## 9. Conclusions

The metastasis formation is a key cause of mortality and the failure of cancer therapy. For the development of metastases, the migratory ability of cancer cells is required. The identification of key molecules in cancer cell migration can open new therapeutic perspectives for successful cancer treatment. In this review, we highlighted the numerous functions of SDC4, a transmembrane proteoglycan, in cell motility and we summarized the recent knowledge about the role of SDC4 in cancer cell movement. Changes in SDC4 expression contribute to cancer growth and progression and have diagnostic and prognostic significance in numerous tumor types. SDC4 modulates several steps in the development and progression of tumors, such as uncontrolled cell proliferation, invasive growth, migration, metastases formation, angiogenesis, as well as tumor-associated inflammation.

Given the ubiquitous expression of SDC4, the summarized SDC4-mediated signaling pathways are likely applicable to several cell types. Importantly, a couple of anticancer drugs modulate SDC4 expression. Because SDC4 has multiple roles in tumor development and progression, targeting SDC4-mediated signaling may be a promising possibility for cancer treatment and drug development; however, the ubiquitous expression of SDC4 would require cancer cell specific targeting.
